# Supplementary material for: Associations of myeloid cells with cellular and humoral responses following vaccinations in patients with neuroimmunological diseases
Source: Nat Commun. 2023 Nov 25;14:7728. doi: 10.1038/s41467-023-43553-z (PMC10676398; doi:10.1038/s41467-023-43553-z)
Supplement: Supplementary file 1 — Supplementary Information [file 41467_2023_43553_MOESM1_ESM.pdf]

Associations of myeloid cells with cellular and humoral responses following vaccinations in patients with neuroimmunological diseases

Meng Wang[1,2,3], Adeline Dehlinger[1,2,3], Camila Fernández Zapata[1,2,3], Maya Golan[4], Gerardina Gallaccio[1,2,3], Leif E. Sander[5], Stephan Schlickeiser[6], Desiree Kunkel[7], Tanja Schmitz-Hübsch[1,2,3,8], Birgit Sawitzki[6], Arnon Karni[4,9], Julian Braun[10,11], Lucie Loyal[10,11], Andreas Thiel[10,11], Judith Bellmann-Strobl[1,2,3], Friedemann Paul[#1,2,3,8,12], Lil Meyer-Arndt[#8,10,12], Chotima Böttcher[✉#1,2,3]

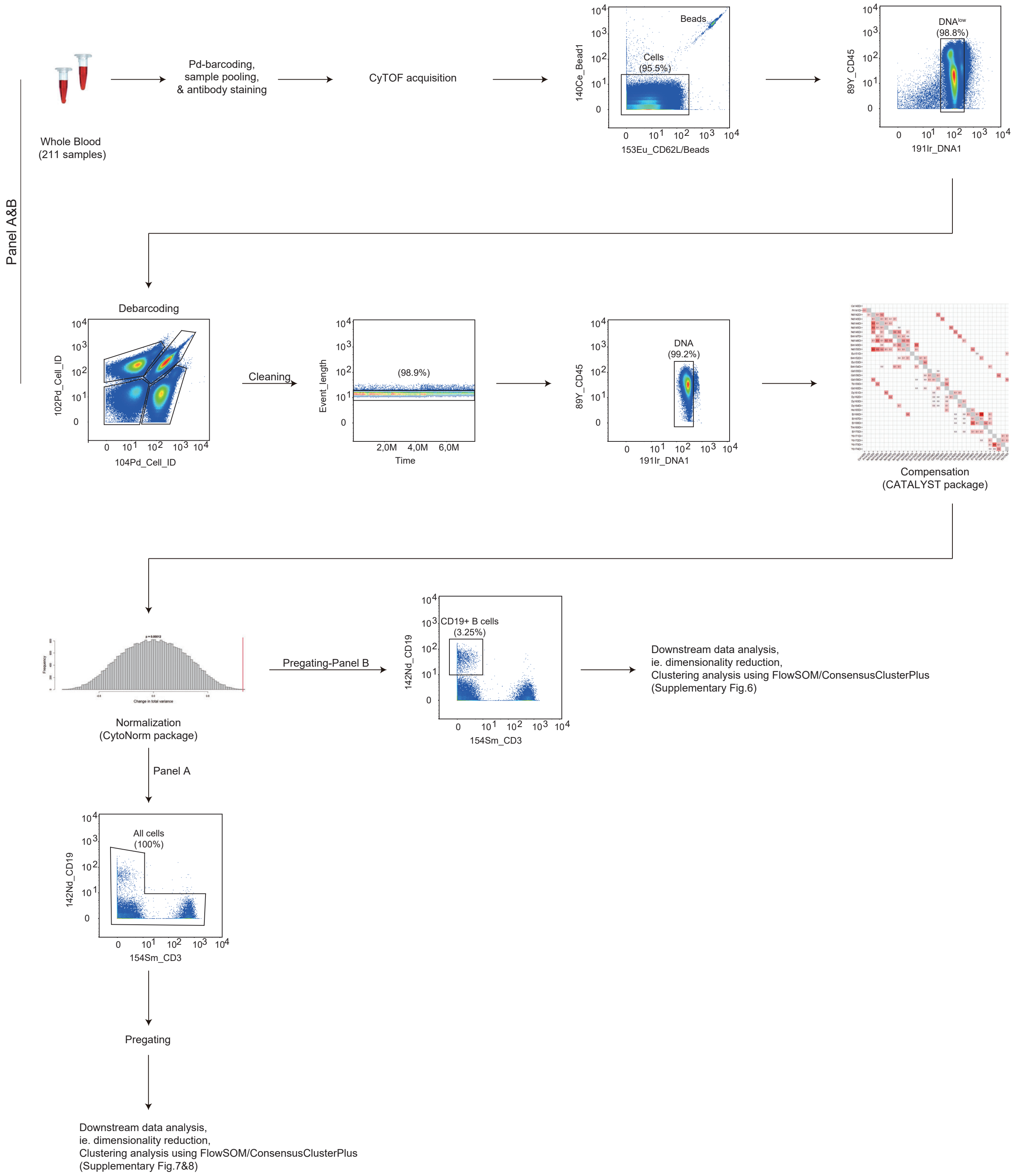
Panel A&B
Whole Blood
(211 samples)
Pd-barcoding,
sample pooling,
& antibody staining
CyTOF acquisition
Beads
Cells
(95.5%)
140Ce_Bead1
153Eu_CD62L/Beads
DNAlow
(98.8%)
89Y_CD45
191Ir_DNA1
Debarcoding
102Pd_Cell_ID
104Pd_Cell_ID
Cleaning
(98.9%)
Event_length
2,0M
4,0M
6,0M
Time
DNA
(99.2%)
89Y_CD45
191Ir_DNA1
Compensation
(CATALYST package)
Normalization
(CytoNorm package)
Pregating-Panel B
CD19+ B cells
(3.25%)
142Nd_CD19
154Sm_CD3
Downstream data analysis,
ie. dimensionality reduction,
Clustering analysis using FlowSOM/ConsensusClusterPlus
(Supplementary Fig.6)
Panel A
All cells
(100%)
142Nd_CD19
154Sm_CD3
Pregating
Downstream data analysis,
ie. dimensionality reduction,
Clustering analysis using FlowSOM/ConsensusClusterPlus
(Supplementary Fig.7&8)

Supplementary Figure 1 Schematic representation of CyTOF measurement and gating strategy from Panel A&B. A total of 211 whole blood samples were collected from MS and NMOSD patients. Whole blood samples were CD45-barcoded and pooled. Mixed samples were equally divided and stained with two panels (Panel A and B, Supplementary Tables 2 and 3) of metal-conjugated antibodies and acquired on the CyTOF instrument. Prior to pre-gating, the data were de-barcoded, compensated and normalized. In Panel A, the gating strategy for selecting single CD45+ cells, excluding CD3+CD19+ double positives. In Panel B, the gating strategy for cell sorting of CD19+ B cells.

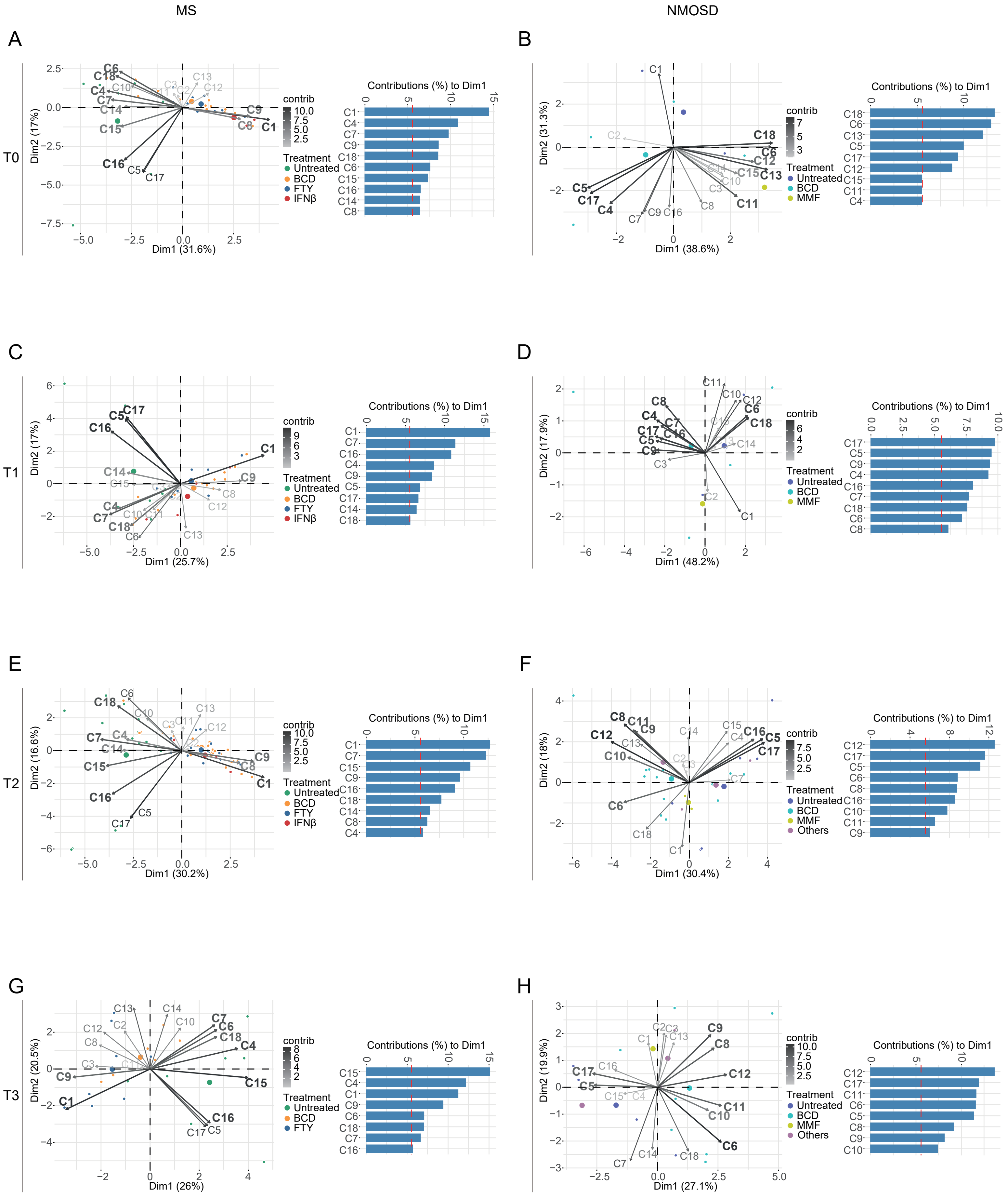
MS
NMOSD
A
B
C
D
E
F
G
H
T0
T1
T2
T3
Dim1 (31.6%)
Dim2 (17%)
Dim1 (38.6%)
Dim2 (31.3%)
Dim1 (25.7%)
Dim2 (17%)
Dim1 (48.2%)
Dim2 (17.9%)
Dim1 (30.2%)
Dim2 (16.6%)
Dim1 (30.4%)
Dim2 (18%)
Dim1 (26%)
Dim2 (20.5%)
Dim1 (27.1%)
Dim2 (19.9%)
Contributions (%) to Dim1
contrib
Treatment
Untreated
BCD
FTY
IFNβ
MMF
Others

Supplementary Figure 2 Contribution of immune cell sub-populations to DMT-mediated differences in cell composition at different timepoints depicted by principal components analysis (PCA)

(A-H) PCA for the 18 identified immune clusters in untreated and DMT-treated MS (A, C, E & G) and NMOSD patients (B, D, F & H) at T0 (A & B), T1 (C & D), T2 (E & F) and T3 (G & H). Each point represents one sample's scores on the first 2 dimensions (Dim1 and Dim2). Each vector (arrow) shows the loadings of each cell cluster on the first 2 principal components. Grey scale indicates the contribution value of variables to Dim1 and Dim2. The red dashed line on the graph indicates the expected average contribution. The graph shows top variables (with a contribution larger than average) contributing to Dim1.

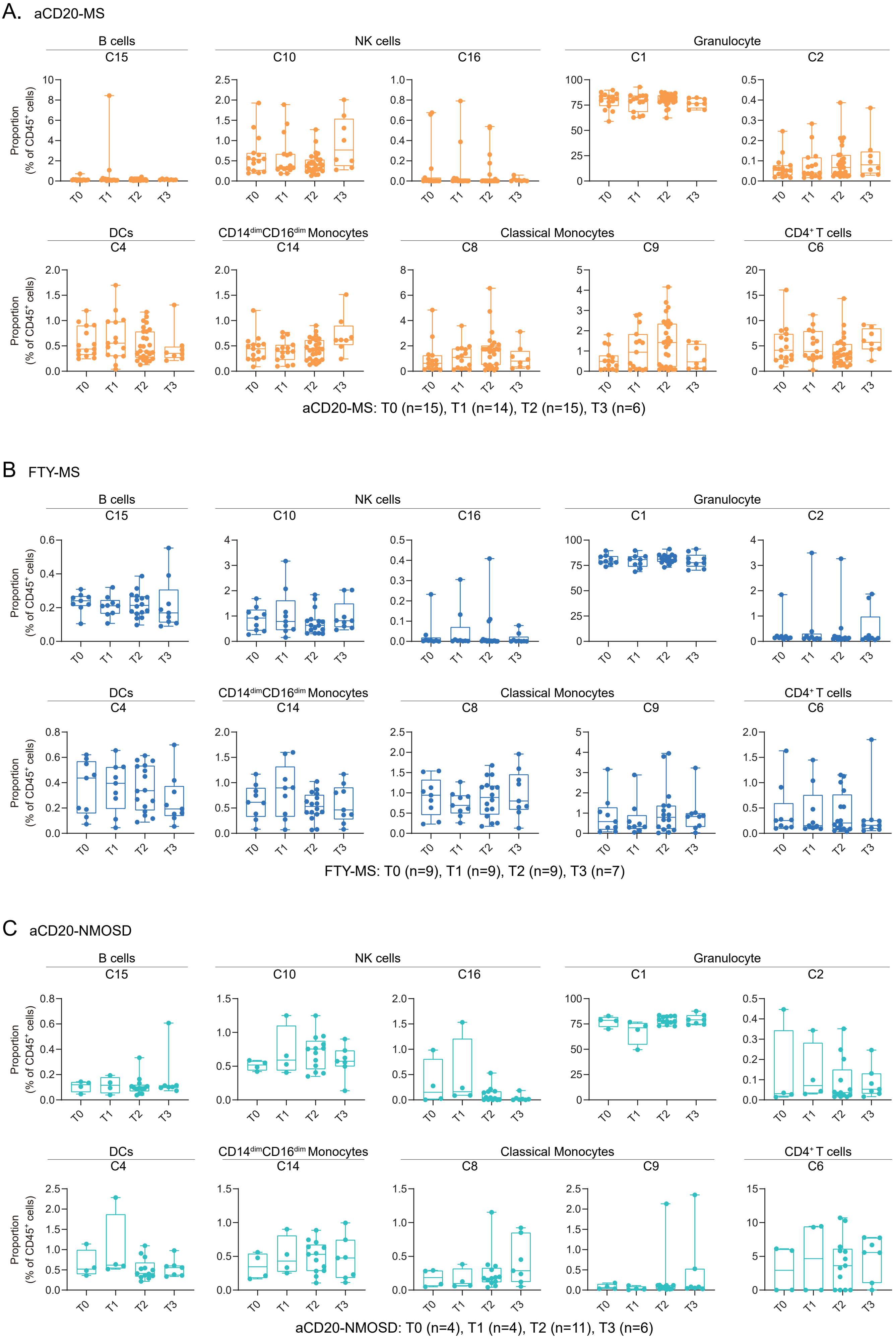
A. aCD20-MS
B cells
C15
NK cells
C10
C16
Granulocyte
C1
C2
DCs
C4
CD14dimCD16dim Monocytes
C14
Classical Monocytes
C8
C9
CD4+ T cells
C6
Proportion (% of CD45+ cells)
T0
T1
T2
T3
aCD20-MS: T0 (n=15), T1 (n=14), T2 (n=15), T3 (n=6)
B FTY-MS
FTY-MS: T0 (n=9), T1 (n=9), T2 (n=9), T3 (n=7)
C aCD20-NMOSD
aCD20-NMOSD: T0 (n=4), T1 (n=4), T2 (n=11), T3 (n=6)

Supplementary Figure 3 Differential proportion of ten defined significant clusters (shown in Fig. 1) between four time points in DMT-treated MS and NMOSD patients.

(A) Box plots show non-significant differences of the ten defined clusters (mean ± SD) between T0 (n=15), T1 (n=14), T2 (n=15), T3 (n=6) in aCD20-treated MS patients (Kruskal-Wallis and Dunn's multiple comparison test). Each dot represents the value of each sample. Boxes extend from the 25th to 75th percentiles. Whisker plots show the min (smallest) and max (largest) values. The line in the box denotes the median.

(B) Box plots show non-significant differences of the ten defined clusters (mean ± SD) between T0 (n=9), T1 (n=9), T2 (n=9), T3 (n=7) in FTY-treated MS patients (Kruskal-Wallis and Dunn's multiple comparison test). Each dot represents the value of each sample. Boxes extend from the 25th to 75th percentiles. Whisker plots show the min (smallest) and max (largest) values. The line in the box denotes the median.

(C) Box plots show non-significant differences of the ten defined clusters (mean ± SD) between T0 (n=4), T1 (n=4), T2 (n=11), T3 (n=6) in aCD20-treated NMOSD patients (Kruskal-Wallis and Dunn's multiple comparison test). Each dot represents the value of each sample. Boxes extend from the 25th to 75th percentiles. Whisker plots show the min (smallest) and max (largest) values. The line in the box denotes the median.

A. T1

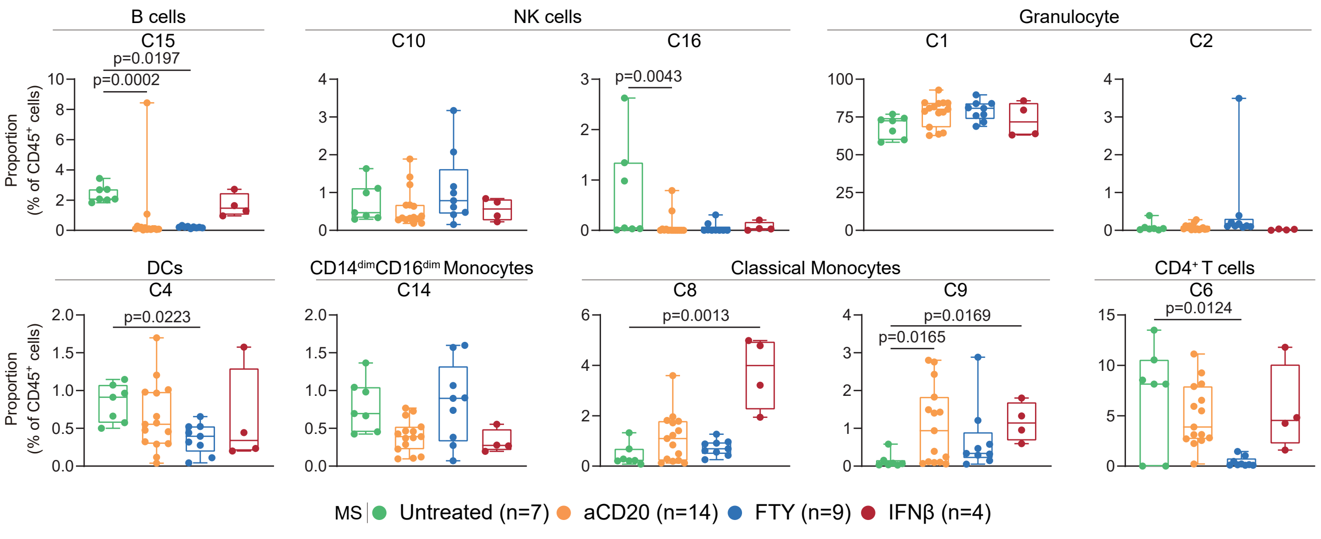
B cells
C15
p=0.0197
p=0.0002
Proportion (% of CD45+ cells)
NK cells
C10
C16
p=0.0043
Granulocyte
C1
C2
DCs
C4
p=0.0223
CD14dimCD16dim Monocytes
C14
Classical Monocytes
C8
p=0.0013
C9
p=0.0169
p=0.0165
CD4+ T cells
C6
p=0.0124
MS Untreated (n=7) aCD20 (n=14) FTY (n=9) IFNβ (n=4)

B T2

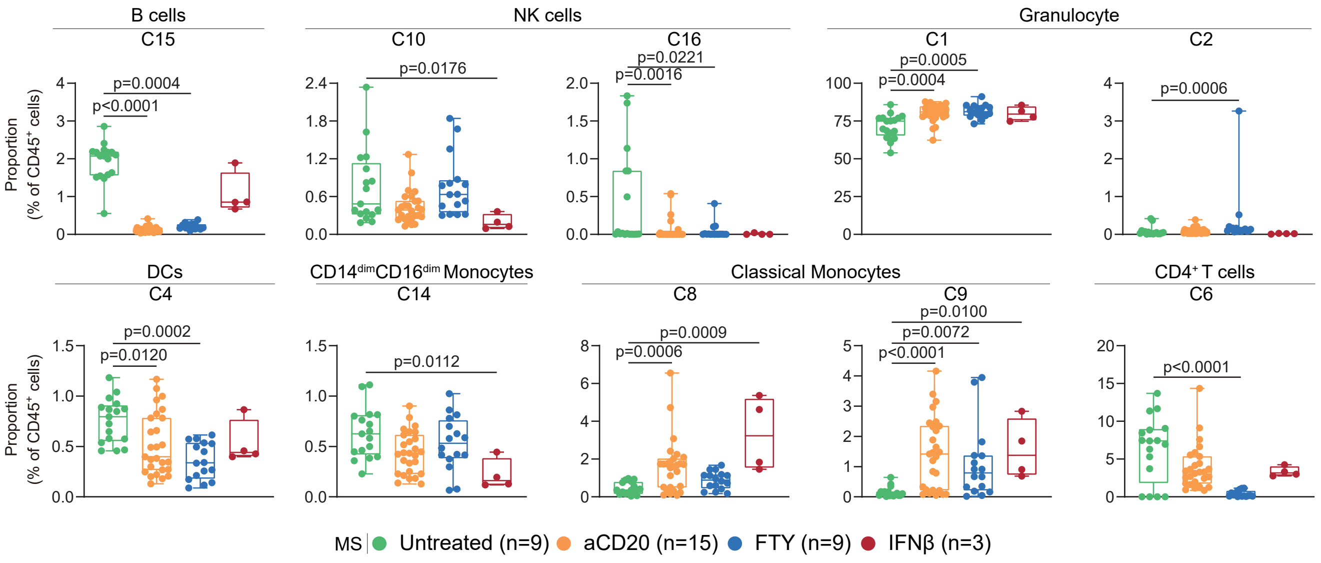
B cells
C15
p=0.0004
p<0.0001
Proportion (% of CD45+ cells)
NK cells
C10
p=0.0176
C16
p=0.0221
p=0.0016
Granulocyte
C1
p=0.0005
p=0.0004
C2
p=0.0006
DCs
C4
p=0.0002
p=0.0120
CD14dimCD16dim Monocytes
C14
p=0.0112
Classical Monocytes
C8
p=0.0009
p=0.0006
C9
p=0.0100
p=0.0072
p<0.0001
CD4+ T cells
C6
p<0.0001
MS Untreated (n=9) aCD20 (n=15) FTY (n=9) IFNβ (n=3)

C T3

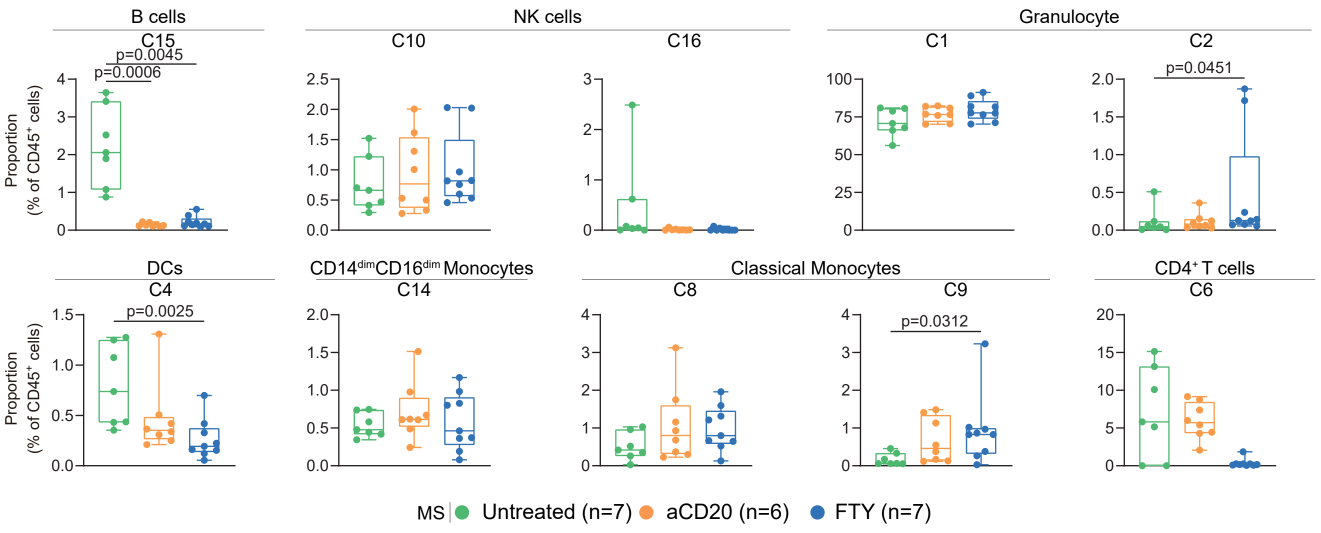
B cells
C15
p=0.0045
p=0.0006
Proportion (% of CD45+ cells)
NK cells
C10
C16
Granulocyte
C1
C2
p=0.0451
DCs
C4
p=0.0025
CD14dimCD16dim Monocytes
C14
Classical Monocytes
C8
C9
p=0.0312
CD4+ T cells
C6
MS Untreated (n=7) aCD20 (n=6) FTY (n=7)

Supplementary Figure 4 Differential proportion of ten defined significant clusters (shown in Fig. 1) between different MS groups at different time points.

(A) Box plots of the ten differentially abundant clusters (mean ± SD) between Untreated-MS (n=7), aCD20-MS (n=14), FTY-MS (n=9), IFNβ-MS (n=4) at T1. Each dot represents the value of each sample. Boxes extend from the 25th to 75th percentiles. Whisker plots show the min (smallest) and max (largest) values. The line in the box denotes the median. Kruskal-Wallis and Dunn's multiple comparison test.

(B) Box plots of the ten differentially abundant clusters (mean ± SD) between Untreated-MS (n=9), aCD20-MS (n=15), FTY-MS (n=9), IFNβ-MS (n=3) at T2. Each dot represents the value of each sample. Boxes extend from the 25th to 75th percentiles. Whisker plots show the min (smallest) and max (largest) values. The line in the box denotes the median. Kruskal-Wallis and Dunn's multiple comparison test.

(C) Box plots of the ten differentially abundant clusters (mean ± SD) between Untreated-MS (n=7), aCD20-MS (n=6), FTY-MS (n=7) at T3. Each dot represents the value of each sample. Boxes extend from the 25th to 75th percentiles. Whisker plots show the min (smallest) and max (largest) values. The line in the box denotes the median. Kruskal-Wallis and Dunn's multiple comparison test.

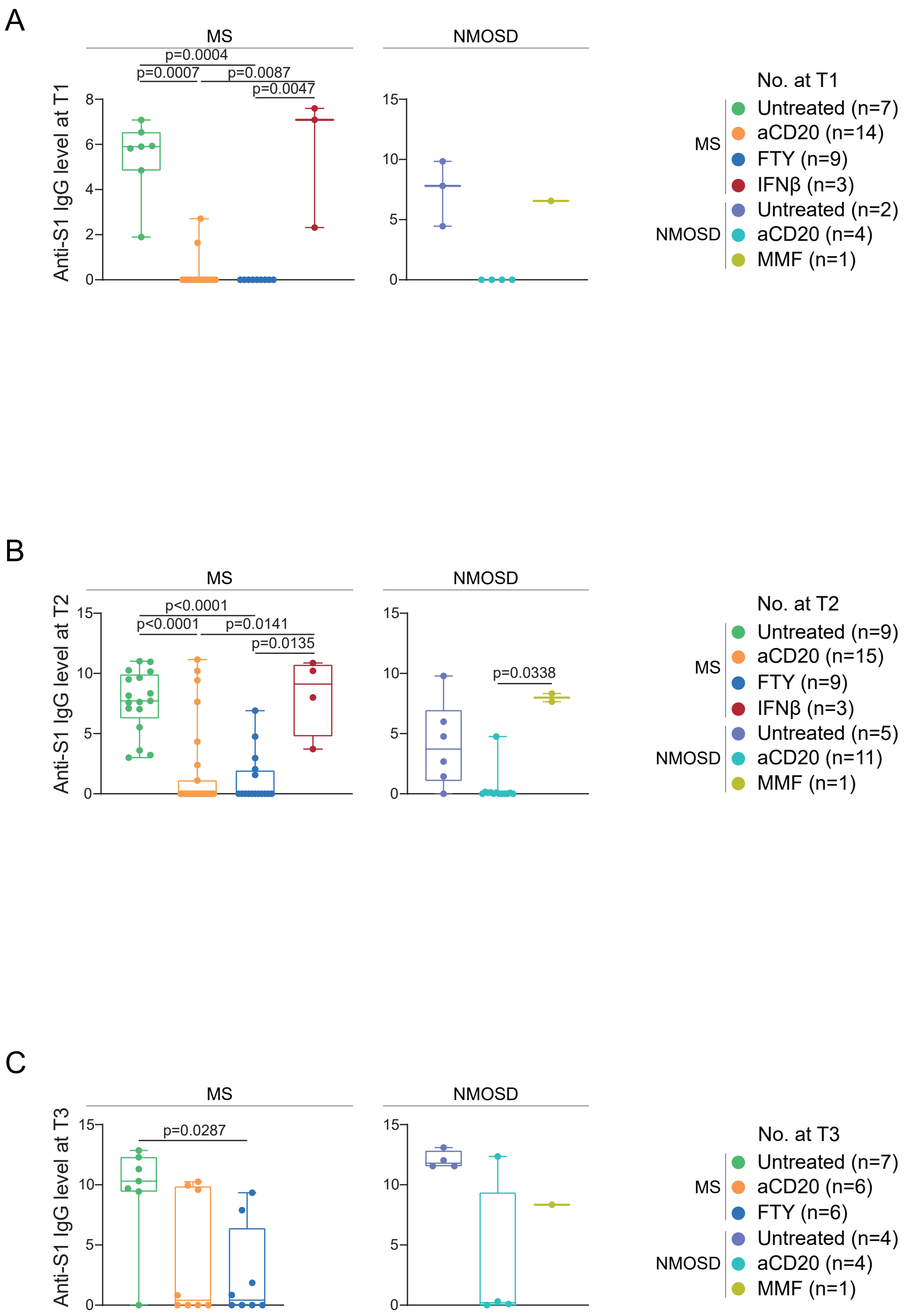
A
MS
NMOSD
p=0.0004
p=0.0007
p=0.0087
p=0.0047
Anti-S1 IgG level at T1
No. at T1
MS
Untreated (n=7)
aCD20 (n=14)
FTY (n=9)
IFNβ (n=3)
NMOSD
Untreated (n=2)
aCD20 (n=4)
MMF (n=1)
B
MS
NMOSD
p<0.0001
p<0.0001
p=0.0141
p=0.0135
p=0.0338
Anti-S1 IgG level at T2
No. at T2
MS
Untreated (n=9)
aCD20 (n=15)
FTY (n=9)
IFNβ (n=3)
NMOSD
Untreated (n=5)
aCD20 (n=11)
MMF (n=1)
C
MS
NMOSD
p=0.0287
Anti-S1 IgG level at T3
No. at T3
MS
Untreated (n=7)
aCD20 (n=6)
FTY (n=6)
NMOSD
Untreated (n=4)
aCD20 (n=4)
MMF (n=1)

Supplementary Figure 5 Humoral response after SARS-CoV-2 mRNA vaccination in patients with MS and NMOSD.

(A) Anti-S1 IgG titer in MS (Untreated-MS (n=7), aCD20-MS (n=14), FTY-MS (n=9), IFNβ-MS (n=3)) and NMOSD (Untreated-NMOSD (n=2), aCD20-NMOSD (n=4), MMF-NMOSD (n=1)) groups at T1. Each dot represents the value of each sample. Boxes extend from the 25th to 75th percentiles. Whisker plots show the min (smallest) and max (largest) values. The line in the box denotes the median. Kruskal-Wallis and Dunn’s multiple comparison test.

(B) Anti-S1 IgG titer in MS (Untreated-MS (n=9), aCD20-MS (n=15), FTY-MS (n=9), IFNβ-MS (n=3)) and NMOSD (Untreated-NMOSD (n=5), aCD20-NMOSD (n=11), MMF-NMOSD (n=1)) groups at T2. Each dot represents the value of each sample. Boxes extend from the 25th to 75th percentiles. Whisker plots show the min (smallest) and max (largest) values. The line in the box denotes the median. Kruskal-Wallis and Dunn’s multiple comparison test.

(C) Anti-S1 IgG titer in MS (Untreated-MS (n=7), aCD20-MS (n=6), FTY-MS (n=6)) and NMOSD (Untreated-NMOSD (n=4), aCD20-NMOSD (n=4), MMF-NMOSD (n=1)) groups at T3. Each dot represents the value of each sample. Boxes extend from the 25th to 75th percentiles. Whisker plots show the min (smallest) and max (largest) values. The line in the box denotes the median. Kruskal-Wallis and Dunn’s multiple comparison test.

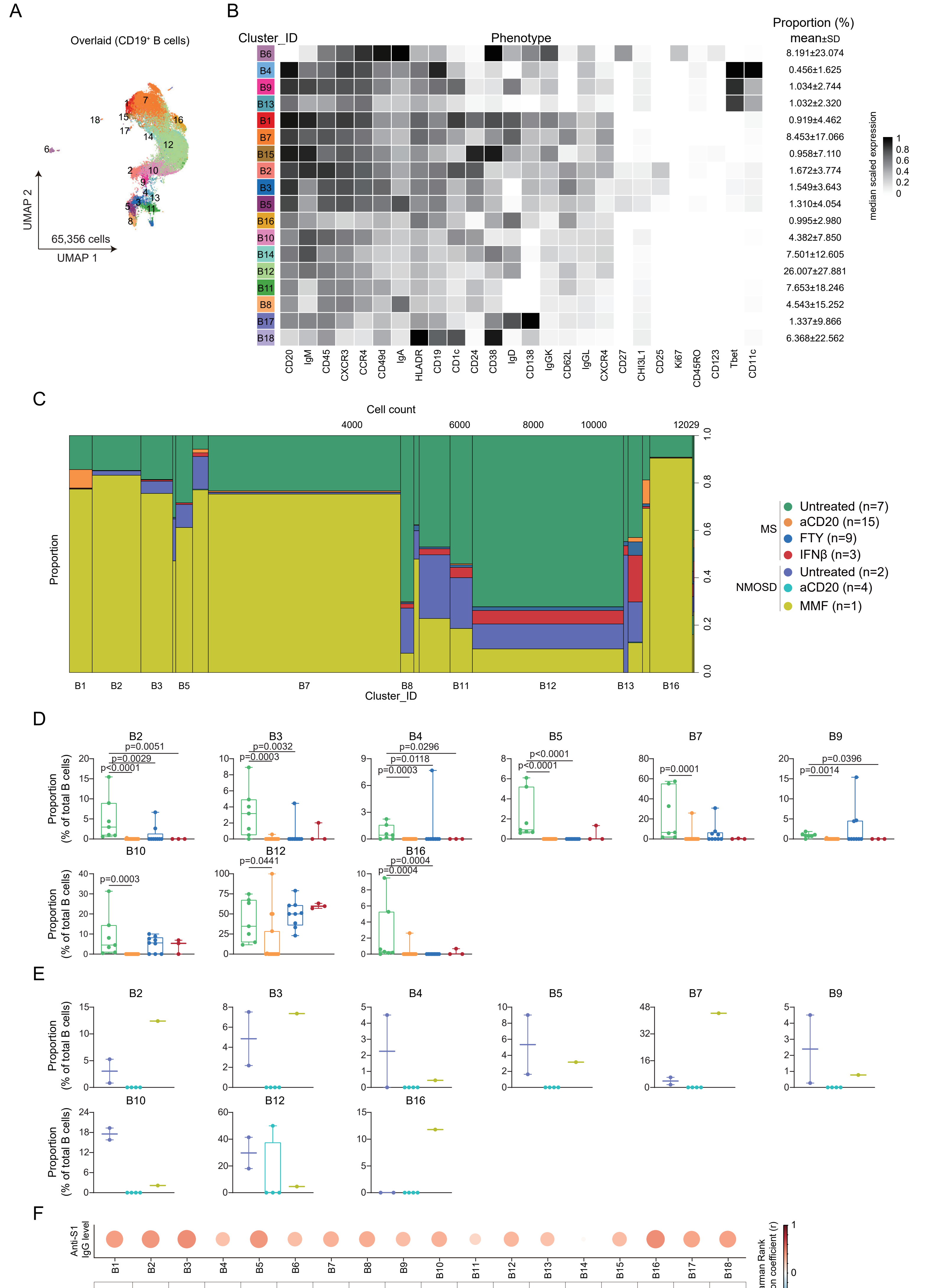

	B1	B2	B3	B4	B5	B6	B7	B8	B9	B10	B11	B12	B13	B14	B15	B16	B17	B18
R	0.3956	0.4242	0.4577	0.2628	0.4233	0.2799	0.3279	0.3415	0.2805	0.3230	0.1713	0.3050	0.2618	0.0207	0.2804	0.4544	0.3689	0.3816
P value	<0.0001	<0.0001	<0.0001	0.0036	<0.0001	0.0019	0.0002	0.0001	0.0018	0.0003	0.0603	0.0007	0.0037	0.8215	0.0018	<0.0001	<0.0001	<0.0001

Supplementary Figure 6 Association of B cell sub-population with anti-S1 IgG antibody production at T1-T3 in MS and NMOSD groups

(A) UMAP plots colored by cluster ID for 1–18 clusters of CD19+ B cells determined using the FlowSOM algorithm.

(B) Phenotypic heatmap depicting the median expression levels of selected markers per B cell cluster.

(C) Mosaic plots depicting cluster proportion and cell count per cluster in each group at T0.

(D) Proportion of the nine differentially abundant clusters (mean ± SD) between Untreated-MS (n=7), aCD20-MS (n=15), FTY-MS (n=9), IFNβ-MS (n=3) at T0. Each dot represents the value of each sample. Boxes extend from the 25th to 75th percentiles. Whisker plots show the min (smallest) and max (largest) values. The line in the box denotes the median. Kruskal-Wallis and Dunn's multiple comparison test.

(E) Proportion of the nine clusters as in D (mean ± SD) between Untreated-NMOSD (n=2), aCD20-NMOSD (n=4), MMF-NMOSD (n=1) at T0. Each dot represents the value of each sample. Boxes extend from the 25th to 75th percentiles. Whisker plots show the min (smallest) and max (largest) values. The line in the box denotes the median.

(F) Heatmap of the Spearman correlation coefficients between the proportion of B cell clusters and antibody titers at T1-T3 in MS groups. Nonparametric Spearman correlation test (r), two-sided.

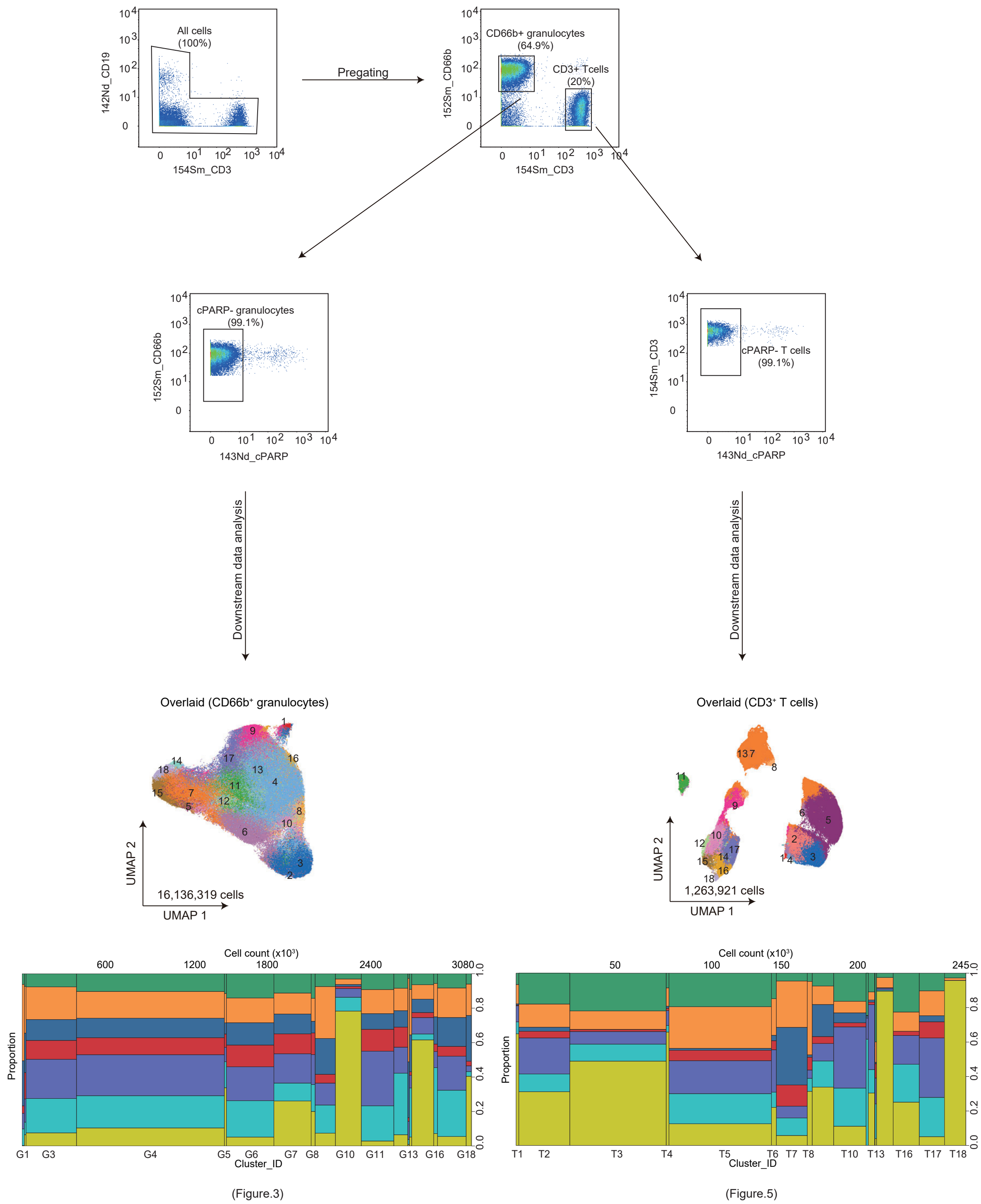

Supplementary Figure 7 Representative plots of gating strategy in Panel A, related to **Figure 3** and **5**. CD66b+ granulocytes and CD3+ T cells were sorted from all CD45+ populations.

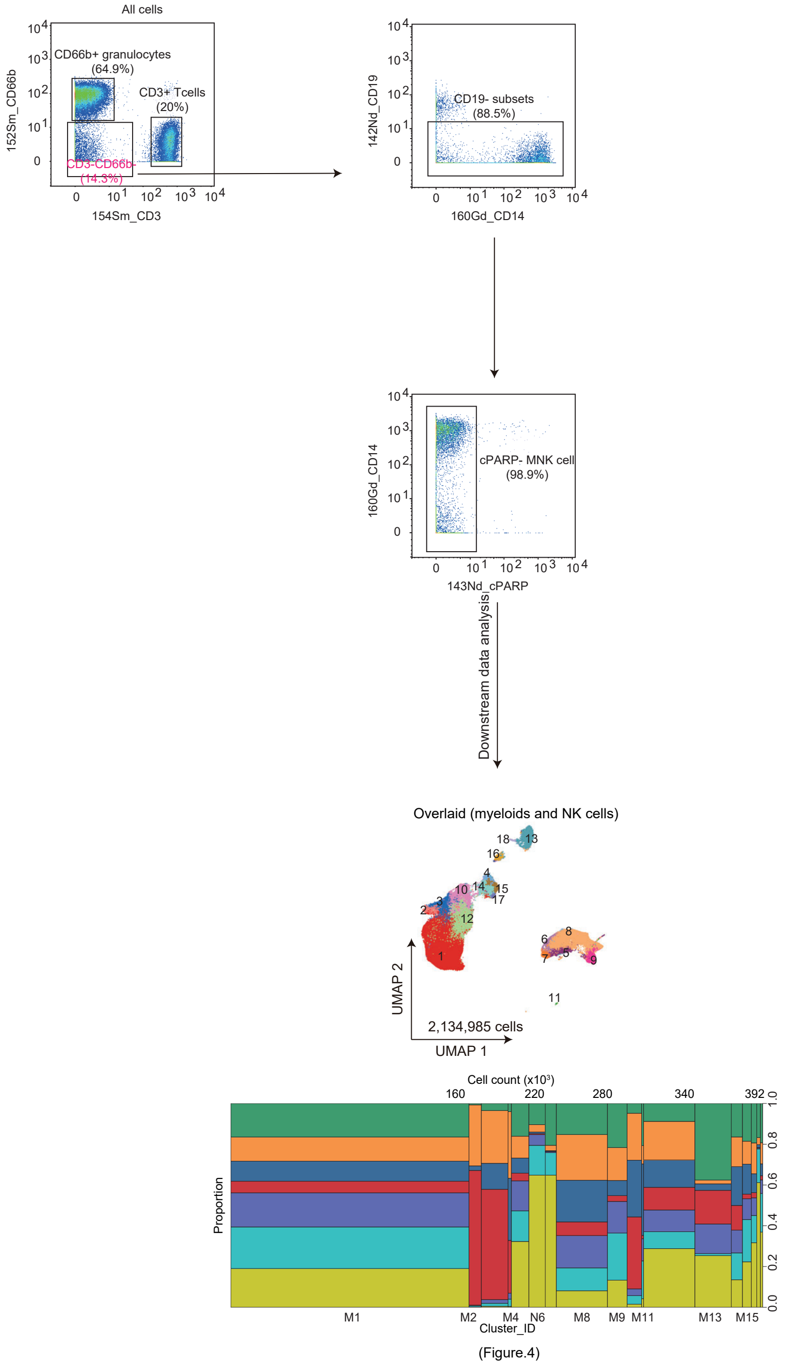

Supplementary Figure 8 Gating strategy for MNK cell in Panel A, used in **Figure 4**. The gating of MNK cells from CD3-CD66b- populations based on marker expression of CD3, CD66b, CD19 and CD14.

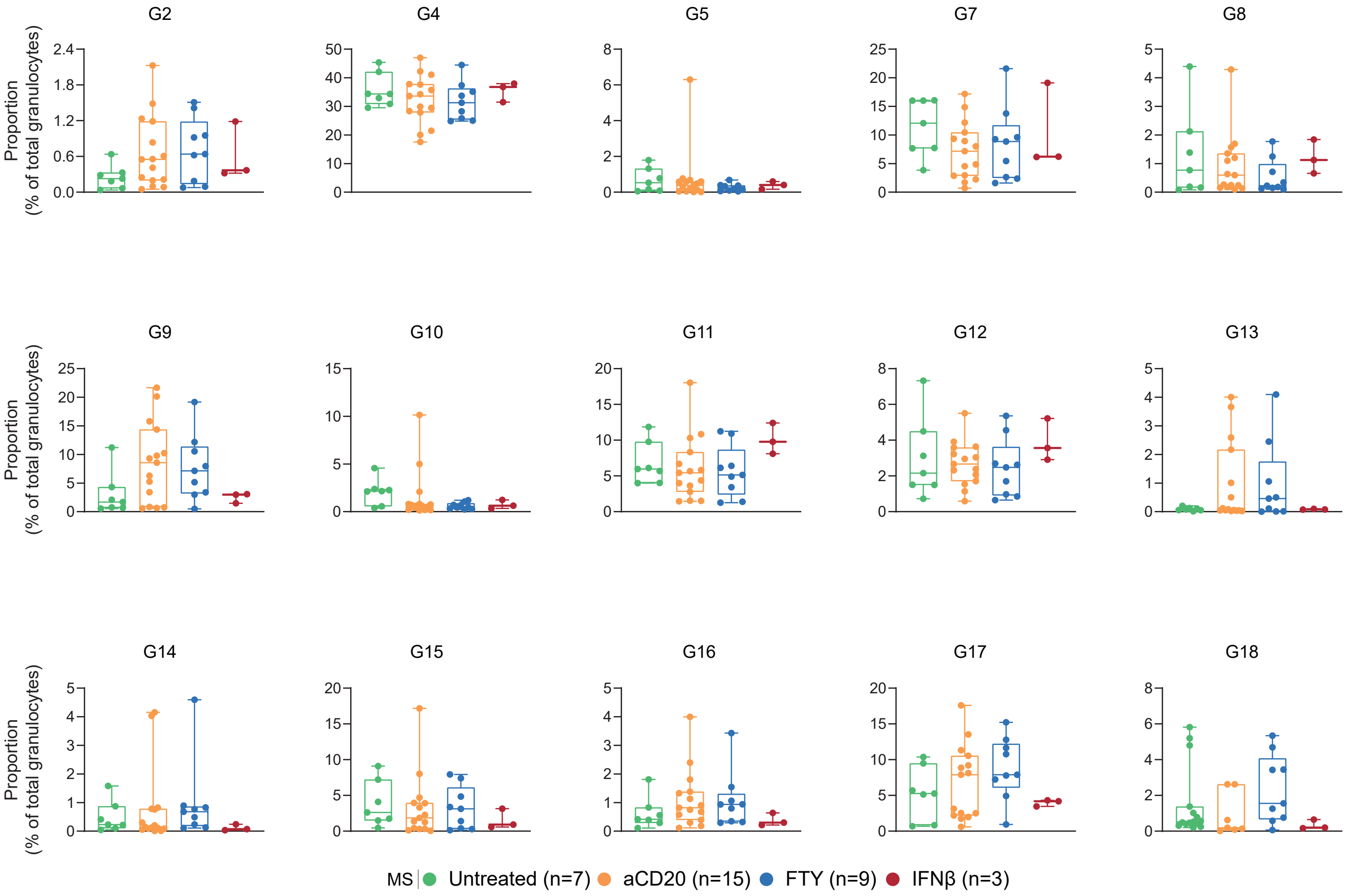

Supplementary Figure 9 Proportion of the fifteen non-significant granulocyte clusters (mean ± SD) between Untreated-MS (n=7), aCD20-MS (n=15), FTY-MS (n=9), IFNβ-MS (n=3) at T0 (Kruskal-Wallis and Dunn's multiple comparison test). Each dot represents the value of each sample. Boxes extend from the 25th to 75th percentiles. Whisker plots show the min (smallest) and max (largest) values. The line in the box denotes the median.

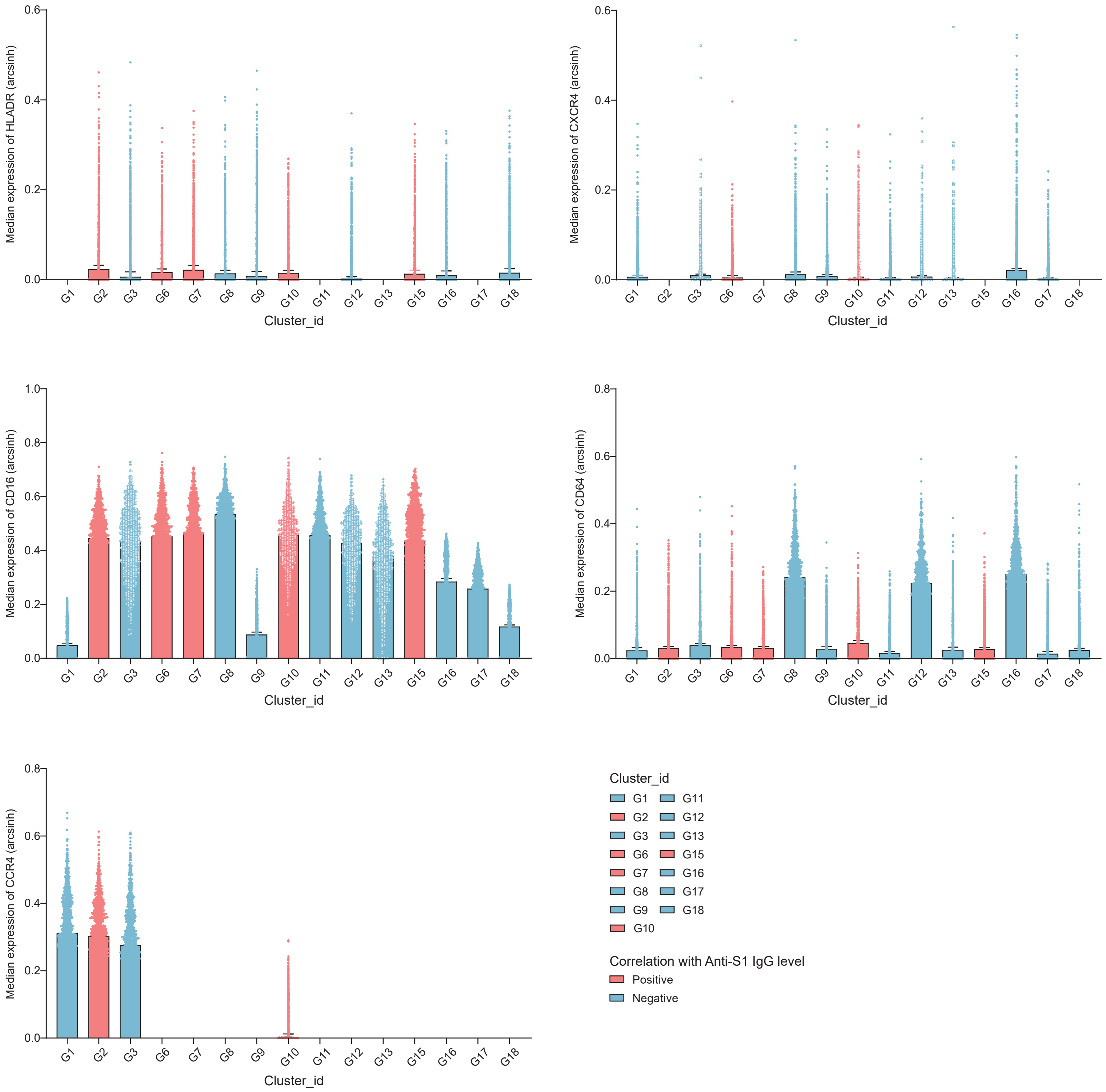

Median expression of HLADR (arcsinh)
Median expression of CXCR4 (arcsinh)
Median expression of CD16 (arcsinh)
Median expression of CD64 (arcsinh)
Median expression of CCR4 (arcsinh)
0.0
0.2
0.4
0.6
0.8
1.0
G1 G2 G3 G6 G7 G8 G9 G10 G11 G12 G13 G15 G16 G17 G18
Cluster_id
Cluster_id
G1 G11
G2 G12
G3 G13
G6 G15
G7 G16
G8 G17
G9 G18
G10
Correlation with Anti-S1 IgG level
Positive
Negative

Supplementary Figure 10 Median expression (with arcsinh transformation) of markers of granulocyte sub-clusters found correlated with anti-S1 IgG antibody levels in MS and NMOSD groups. Each dot represents the value of each single cell. Data displayed as median with 95% confidence interval. The red bars represent the clusters that show positive correlations with anti-S1 IgG antibody levels. The light blue bars represent the clusters that show negative correlations with anti-S1 IgG antibody levels.

Supplementary Table 1. Patients information

Group	MS	NMOSD
**total no.**	39	23
**Female/Male**	29/10	18/5
**Age, mean±SD (range, years)**	48±11 (28-69)	48±15 (21-78)
**Therapy (no.)**		
**Untreated**	9	5
**aCD20**	16	13
**FTY**	9	-
**IFNβ**	5	-
**AZA**	-	2
**MMF**	-	1
**TCZ**	-	1
**IVIG**	-	1
**Timepoint (patient no./sample no.)**		
**T0**	34/34	7/7
**T1**	34/35*	7/7
**T2**	36/64**	20/24**
**T3**	20/24***	13/16***
**Disease duration, mean±SD (range, years)**	20±9 (3-42)	11±7 (2-37)
**Treatment duration before vaccination, mean±SD (range, months)**	44±50 (2-233)	68±53 (17-155)
**EDSS, mean±SD (range)**		
**at T0**	3.1±1.8 (0-7)	3.4±1.9 (2-6)
**at T1**	3.3±1.4 (1.5-5)	4.8±1.8 (3.5-6)
**at T2**	3.1±1.6 (0-6)	2.5±1.9 (0-6.5)
**at T3**	3±0.5 (2.5-3.5)	1.7±0.6 (1-2)

* 2 patients have 2 samples at different timepoints after primary but before secondary vaccination.
** 31 patients have 2 or 3 samples at different timepoints but within 6 months after secondary vaccination.
*** 5 patients have 2 or 3 samples at different timepoints but within 4 months after tertiary vaccination.
Abbreviations: MS= multiple sclerosis; NMOSD= neuromyelitis optica spectrum disorders; aCD20= anti-CD20 monoclonal antibodies; FTY= fingolimod; IFNβ= interferon β; AZA= azathioprine; MMF= mycophenolate mofetil; TCZ= tocilizumab; IVIG= Intravenous immunoglobulin.

Supplementary Table 2. The *Panel A*-antibody list

Target	Isotope tag	clone	company	Dilution
**Surface**				
CD45	^{89}Y	HI30	Fluidigm	1:200
HLA-DR	^{141}Pr	L243	Biolegend	1:100
CD19	^{142}Nd	HIB19	Fluidigm	1:100
CD69	^{144}Nd	FN50	Fluidigm	1:100
CD4	^{145}Nd	RPA-T4	Fluidigm	1:100
CD64	^{146}Nd	10.1	Fluidigm	1:100
CD226	^{147}Sm	REA1040	Miltenyi	1:100
CD16	^{148}Nd	3G8	Fluidigm	1:50
CD56	^{149}Sm	NCAM16.2	Fluidigm	1:400
ICOS	^{151}Eu	C398.4A	Fluidigm	1:50
CD66b	^{152}Sm	80H3	Fluidigm	1:600
CD3	^{154}Sm	UCHT1	Fluidigm	1:200
CD11c	^{155}Gd	Bu15	Biolegend	1:200
CCR4	^{158}Gd	L291H4	Fluidigm	1:200
TIGIT	^{159}Tb	MBSA43	Fluidigm	1:50
CD14	^{160}Gd	RM052	Fluidgm	1:100
CD8	^{162}Dy	RPA-T8	Fluidigm	1:50
CRTH2	^{163}Dy	BM16	Fluidigm	1:100
CD95	^{164}Dy	DX2	Fluidigm	1:50
LAG3	^{165}Ho	11C3C65	Fluidigm	1:100
CD141	^{166}Er	M80	Fluidigm	1:50
CCR7	^{167}Er	G043H7	Fluidigm	1:100
CD206	^{168}Er	15-2	Fluidigm	1:50
CD33	^{169}Tm	WM53	Fluidigm	1:100
FceR1a	^{170}Er	AER-37 (CRA1)	eBioscience	1:100
CD161	^{171}Yb	HP-3G10	Biolegend	1:100
CXCR4	^{173}Yb	12G5	Fluidigm	1:200
CD127	^{176}Yb	A019D5	DVS Sciences	1:100
CD47	^{209}Bi	CC2C6	Fluidigm	1:200
**Intracellular**				
cPARP	^{143}Nd	F21-852	fluidigm	1:200
MIP1beta	^{150}Nd	D21-1351	Fluidigm	1:100
CD68	^{153}Eu	Y1/82A	Biolegend	1:100
IL6	^{156}Gd	MQ2-13AS	Fluidigm	1:50
CTLA4	^{161}Dy	14D3	Fluidigm	1:50
OPN	^{172}Yb	polyclonal	LSBio	1:50
IL1beta	^{174}Yb	CRM56	eBioscience	1:50
TNF	^{175}Lu	MAb11	DVS Sciences	1:50

Supplementary Table 3. The *Panel B*-antibody list

Target	Isotope tag	clone	company	Dilution
**Surface**				
CD45	^{89}Y	HI30	Fluidigm	1:200
CD49d	^{141}Pr	9F10	Fluidigm	1:100
CD19	^{142}Nd	HIB19	Fluidigm	1:100
HLA-DR	^{143}Nd	L243	Fluidigm	1:200
CD38	^{144}Nd	HIT2	Fluidigm	1:100
CD138	^{145}Nd	DL-101	Fluidigm	1:200
IgD	^{146}Nd	IA6-2	Fluidigm	1:100
CD123	^{147}Sm	6H6	Biolegend	1:100
IgA	^{148}Nd	polyclonal	Fluidigm	1:200
CD25	^{149}Sm	2A3	Fluidigm	1:50
KLRG1	^{150}Nd	14C2A07	Biolegend	1:100
IgGL	^{151}Eu	MHL-38	Fluidigm	1:200
TCRgd	^{152}Sm	11F2	Fluidigm	1:100
CD62L	^{153}Eu	DREG-56	Fluidigm	1:100
CD3	^{154}Sm	UCHT1	Fluidigm	1:200
CD11c	^{155}Gd	Bu15	Biolegend	1:100
CXCR3	^{156}Gd	G025H7	Fluidigm	1:200
CCR4	^{158}Gd	L291H4	Fluidigm	1:200
CD1c	^{159}Tb	L161	Biolegend	1:100
IgGK	^{160}Gd	MHK-49	Fluidigm	1:100
CD8	^{162}Dy	RPA-T8	Fluidigm	1:50
CRTH2	^{163}Dy	BM16	Fluidigm	1:100
CD28	^{164}Dy	L293	BDBioscience	1:50
CD45RO	^{165}Ho	UCHL1	Fluidigm	1:100
CD34	^{166}Er	581	Fluidgm	1:100
CD27	^{167}Er	O323	Fluidigm	1:50
CD24	^{169}Tm	ML5	Fluidigm	1:50
CD20	^{171}Yb	2H7	Fluidigm	1:100
IgM	^{172}Yb	MHM-88	Fluidigm	1:100
CXCR4	^{173}Yb	12G5	Fluidigm	1:200
PD1	^{174}Yb	EH12.2H7	Fluidigm	1:50
CD11b	^{209}Bi	ICRF44	Fluidigm	1:200
**Intracellular**				
CTLA4	^{161}Dy	14D3	Fluidigm	1:50
Tbet	^{170}Er	4B10	Biolegend	1:50
Ki67	^{168}Er	B56	Fluidigm	1:50
TNF	^{175}Lu	MAb11	DVS Sciences	1:50
CHI3L1	^{176}Yb	EPR19078-157	abcam	1:100

Supplementary Table 4. Selected markers used in the clustering of All cell, T cell, granulocyte and MNK cell-*Panel A*

**Target in All cells**	**Target in T cells**	**Target in granulocytes**	**Target in MNK cells**
FceR1a	CD3	FceR1a	CD47
CCR4	CCR4	CD45	HLADR
CD11c	CD4	CD95	CCR4
CD68	CCR7	CD66b	CD11c
HLADR	CD8	CD16	CD68
CD64	CRTH2	CD64	CD14
CD14	CTLA4	CCR4	CD33
CD33	CD226	CD69	CD4
CD3	ICOS	CD161	CCR7
CD4	TIGIT	CXCR4	CD64
CD8	CD127	HLADR	CD95
CD66b	CD69	IL1beta	CD206
CD161	LAG3	CD33	CD16
CD56		CD14	OPN
CD19			CD69
			CD161
			CD56
			CD226
			TIGIT
			CD8
			CRTH2
			CD141
			IL1beta
			CD127
			TNF
			MIP1beta

Supplementary Table 5. Selected markers used in B cell subclustering-*Panel B*

**Target**	**Isotope tag**	**clone**	**company**
CD20	^{171}Yb	2H7	Fluidigm
IgM	^{172}Yb	MHM-88	Fluidigm
CXCR3	^{156}Gd	G025H7	Fluidigm
CCR4	^{158}Gd	L291H4	Fluidigm
CD45	^{89}Y	HI30	Fluidigm
CD49d	^{141}Pr	9F10	Fluidigm
HLADR	^{143}Nd	L243	Fluidigm
CD19	^{142}Nd	HIB19	Fluidigm
CD1c	^{159}Tb	L161	Biolegend
CD38	^{144}Nd	HIT2	Fluidigm
IgA	^{148}Nd	polyclonal	Fluidigm
CD138	^{145}Nd	DL-101	Fluidigm
IgD	^{146}Nd	IA6-2	Fluidigm
IgGK	^{160}Gd	MHK-49	Fluidigm
IgGL	^{151}Eu	MHL-38	Fluidigm
CD62L	^{153}Eu	DREG-56	Fluidigm
CD24	^{169}Tm	ML5	Fluidigm
KLRG1	^{150}Nd	14C2A07	Biolegend
CXCR4	^{173}Yb	12G5	Fluidigm
CHI3L1	^{176}Yb	EPR19078-157	abcam
CD25	^{149}Sm	2A3	Fluidigm
CD123	^{147}Sm	6H6	Biolegend
CD45RO	^{165}Ho	UCHL1	Fluidigm
TCRgd	^{152}Sm	11F2	Fluidigm
CD27	^{167}Er	O323	Fluidigm
Ki67	^{168}Er	B56	Fluidigm
CD11c	^{155}Gd	Bu15	Biolegend
Tbet	^{170}Er	4B10	Biolegend

Supplementary Table 6. Treatment duration in MS and NMOSD patients

MS_aCD20 (n=14, months)	MS_FTY (n=8, months)	MS_IFNs (n=3, months)	NMOSD_aCD20 (n=4, months)	NMOSD_MMF (n=1, months)
31	11	233	17	75
15	29	70	34	
11	45	34	155	
2	128		60	
30	123			
47	34			
29	10			
40	71			
25				
4				
9				
35				
17				
22				
