# Supplementary material for: Associations of myeloid cells with cellular and humoral responses following vaccinations in patients with neuroimmunological diseases
Source: Nat Commun. 2023 Nov 25;14:7728. doi: 10.1038/s41467-023-43553-z (PMC10676398; doi:10.1038/s41467-023-43553-z)
Supplement: Supplementary file 3 — Reporting Summary [file 41467_2023_43553_MOESM3_ESM.pdf]

nature portfolio

Corresponding author(s): Chotima Böttcher

Last updated by author(s): Oct 30, 2023

Reporting Summary

Nature Portfolio wishes to improve the reproducibility of the work that we publish. This form provides structure for consistency and transparency in reporting. For further information on Nature Portfolio policies, see our Editorial Policies and the Editorial Policy Checklist.

Statistics

For all statistical analyses, confirm that the following items are present in the figure legend, table legend, main text, or Methods section.

n/a	Confirmed	
☐	☒	The exact sample size (*n*) for each experimental group/condition, given as a discrete number and unit of measurement
☐	☒	A statement on whether measurements were taken from distinct samples or whether the same sample was measured repeatedly
☐	☒	The statistical test(s) used AND whether they are one- or two-sided<br>*Only common tests should be described solely by name; describe more complex techniques in the Methods section.*
☐	☒	A description of all covariates tested
☐	☒	A description of any assumptions or corrections, such as tests of normality and adjustment for multiple comparisons
☐	☒	A full description of the statistical parameters including central tendency (e.g. means) or other basic estimates (e.g. regression coefficient) AND variation (e.g. standard deviation) or associated estimates of uncertainty (e.g. confidence intervals)
☐	☒	For null hypothesis testing, the test statistic (e.g. *F*, *t*, *r*) with confidence intervals, effect sizes, degrees of freedom and *P* value noted<br>*Give P values as exact values whenever suitable.*
☒	☐	For Bayesian analysis, information on the choice of priors and Markov chain Monte Carlo settings
☐	☒	For hierarchical and complex designs, identification of the appropriate level for tests and full reporting of outcomes
☐	☒	Estimates of effect sizes (e.g. Cohen's *d*, Pearson's *r*), indicating how they were calculated

Our web collection on statistics for biologists contains articles on many of the points above.

Software and code

Policy information about availability of computer code

Data collection	CyTOF acquisition software 6.5.236
Data analysis	FlowJo software 10.4.2; R/Bioconductor packages<br>Codes used for CyTOF data analysis in this study are previously published by Crowell H et al. 2022 and available on https://github.com [https://github.com/HelenaLC/CATALYST].

For manuscripts utilizing custom algorithms or software that are central to the research but not yet described in published literature, software must be made available to editors and reviewers. We strongly encourage code deposition in a community repository (e.g. GitHub). See the Nature Portfolio guidelines for submitting code & software for further information.

Data

Policy information about availability of data

All manuscripts must include a data availability statement. This statement should provide the following information, where applicable:

- Accession codes, unique identifiers, or web links for publicly available datasets
- A description of any restrictions on data availability
- For clinical datasets or third party data, please ensure that the statement adheres to our policy

Source data are enclsoed. The raw data are available at https://flowrepository.org/id/FR-FCM-Z7ZZ

Research involving human participants, their data, or biological material

Policy information about studies with human participants or human data. See also policy information about sex, gender (identity/presentation), and sexual orientation and race, ethnicity and racism.

Reporting on sex and gender	The information about sex and gender of the patients in this study was addressed in the Supplementary Table 1. The predominance of females among patients with neuroimmunological diseases such as multiple sclerosis (MS) and neuromyelitis optica spectrum disorders (NMOSD) is well recognized. In our study, there are more female than male patients with MS and NMOSD. Due to limitation of sample size, we could not rule out sex and gender effects on our finding.
Reporting on race, ethnicity, or other socially relevant groupings	since we recruited only caucasian patients who live in Germany, we did not control for confounding regarding race and ethnicity.
Population characteristics	see Supplementary Table 1
Recruitment	see Materials and Methods "Study design" (page 16-17); see Supplementary Table 1
Ethics oversight	The data analyzed here was collected as part of the CCC study at the Charité – Universitätsmedizin Berlin, which was approved by the ethics committee of the Charité–Universitätsmedizin Berlin (Ethikkommission der Charité–Universitätsmedizin Berlin; registration number EA2/224/21) in accordance with the Declaration of Helsinki of 1964 and its later amendments. Patients were recruited via the Charité's MS outpatient clinic. All study participants provided informed consent before any study-related procedures were undertaken and did not receive a compensation.

Note that full information on the approval of the study protocol must also be provided in the manuscript.

Field-specific reporting

Please select the one below that is the best fit for your research. If you are not sure, read the appropriate sections before making your selection.

☒ Life sciences ☐ Behavioural & social sciences ☐ Ecological, evolutionary & environmental sciences

For a reference copy of the document with all sections, see nature.com/documents/nr-reporting-summary-flat.pdf

Life sciences study design

All studies must disclose on these points even when the disclosure is negative.

Sample size	No priori statistical methods were used to predetermine sample sizes due to sample accessibility and insufficient previous data (e.g. phenotypic changes of granulocytes after vaccination) to enable this. However, sample sizes were chosen based on estimates of anticipated variability through our previous studies on similar topic (i.e., Meyer-Arndt, L. et al., J Neurol Neurosurg Psychiatry. 93, 960-971 (2022)) .
Data exclusions	The filtering parameters for differential analysis of cell population abundance was set to minimum number of cells = 3 in at least minimum number of samples = number of samples in each group. Only samples with more than 50 cells were considered for clustering analysis. Of note the inclusion and exclusion criteria of the study were described in the "study design" on page 16-17.
Replication	For CyTOF measurement, some marker expression of each individual samples was measured twice (antibody panel A and B) and were reliably reproduced. For IgG antibody and CD4 activation, we remeasured several donors (accompanied by anti-SARS-CoV-2 IgG antibody testing) at later timepoints and used another Spike glycoprotein peptide pool from Miltenyi to ensure reproducibility of T cell reactivity in SARS-CoV-2 naive donors.
Randomization	no method of randomization was used in this study, since it was not appicable to this study.
Blinding	No blinding was done, since we performed unsupervised data processing and data analysis, thus excluded the possibility of biased results.

Reporting for specific materials, systems and methods

We require information from authors about some types of materials, experimental systems and methods used in many studies. Here, indicate whether each material, system or method listed is relevant to your study. If you are not sure if a list item applies to your research, read the appropriate section before selecting a response.

Materials & experimental systems

n/a	Involved in the study
☐	☒ Antibodies
☒	☐ Eukaryotic cell lines
☒	☐ Palaeontology and archaeology
☒	☐ Animals and other organisms
☒	☐ Clinical data
☒	☐ Dual use research of concern
☒	☐ Plants

Methods

n/a	Involved in the study
☒	☐ ChIP-seq
☒	☐ Flow cytometry
☒	☐ MRI-based neuroimaging

Antibodies

Antibodies used

Panel A:
CD45 (clone HI30, cat# 3089003B) Fluidigm,
HLA-DR (clone L243, cat# 307651) Biolegend,
CD19 (clone HIB19, cat# 3142001B) Fluidigm,
CD69 (clone FN50, cat# 3144018B) Fluidigm,
CD4 (clone RPA-T4, cat# 3145001B) Fluidigm,
CD64 (clone 10.1, cat# 3146006B) Fluidigm,
CD226 (clone REA1040, cat# 130-126-485) Miltenyi,
CD16 (clone 3G8, cat# 3165001B) Fluidigm,
CD56 (clone NCAM16.2, cat# 3149021B) Fluidigm,
ICOS (clone C398.4A, cat# 3151020B) Fluidigm,
CD66b (clone 80H3, cat# 3152011B) Fluidigm,
CD3 (clone UCHT1, cat# 3154003B) Fluidigm,
CD11c (clone 3.9, cat# 3146014B) Fluidigm,
CCR4 (clone L291H4, cat# 3158032A) Fluidigm,
TIGIT (clone MBSA43, cat# 3159038B) Fluidigm,
CD14 (clone RM052, cat# 3160006B) Fluidigm,
CTLA-4 (clone 14D3, cat# 3161004B) Fluidigm,
CD8 (clone RPA-T8, cat# 3162015B) Fludigim,
CRTH2(clone BM16, cat# 3163003B) Fludigim,
CD95 (clone DX2, cat# 3152017B) Fluidigm,
LAG-3(clone 11C3C65, cat# 3165037B) Fluidigm,
CD141 (clone 1A4, cat# 3173002B) Fluidigm,
CCR7 (clone G043H7, cat# 3167009A) Fluidigm,
CD206 (clone 15-2, cat# 3168008B) Biolegend,
CD33 (clone WM53, cat# 3169010B) Fluidigm,
FceR1a (clone AER-37 (CRA1), cat# 13-5899-82) eBioscience,
CD161 (clone HP-3G10, cat# 339919) Biolegend,
CXCR4 (clone 12G5, cat# 3173001B) Fluidigm,
CD127 (clone A019D5, cat# 3176004B) Fluidigm,
CD47 (clone CC2C6, cat# 3209004B) Fluidigm,
cPARP (clone F21-852, cat# 3143011A) Fluidigm,
MIP-1β/CCL4 (clone D211351, cat# 3150004B) Fluidigm,
CD68 (clone Y1/82A, cat# 333802) Biolegend,
IL-6 (clone MQ2-13A5, cat# 501115) Biolegend,
OPN (polyclonal, cat# C99283) LSBio,
IL-1β (clone CRM56, cat# 14-7018-85) eBioscience,
TNF (clone MAb11, cat# 3175023B) DVS Sciences,

Panel B:

CD45 (clone HI30, cat# 3089003B) Fluidigm,
CD49d (clone 9F10, cat# 3141004B) Fluidigm,
CD19 (clone HIB19, cat# 3142001B) Fluidigm,
HLA-DR (clone L243, cat# 307651) Biolegend,
CD38 (clone HIT2, cat# 3167001B) Fluidigm,
CD138 (clone DL-101, cat# 3145003B) Fluidigm,
IgD (clone IA6-2, cat# 3146005B) Fluidigm,
CD123 (clone 6H6, cat# 306002) Biolegend,
IgA (clone polyclonal, cat# 3148007B) Fluidigm,
CD25 (clone 2A3, cat# 3149010B) Fluidigm,
KLRG1 (clone 14C2A07, cat# 368602) Biolegend,
IgGL (clone MHL-38, cat# 3151004B) Fluidigm,
TCRgd (clone 11F2, cat# 3152008B) Fluidigm,
CD62L (clone DREG-56, cat# 3153004B) Fluidigm,
CD3 (clone UCHT1, cat# 3154003B) Fluidigm,
CD11c (clone Bu15, cat# 3147008B) Fluidigm,
CXCR3 (clone G025H7, cat# 3163004B) Fluidigm,

CCR4 (clone L291H4, cat# 3158032A) Fluidigm,
CD1c (clone L161, cat# 331502) Biolegend,
IgGK (clone MHK-49, cat# 3160005B) Fluidigm,
CTLA-4 (clone 14D3, cat# 3161004B) Fluidigm,
CD8 (clone RPA-T8, cat# 3162015B) Fludigim,
CRTH2(clone BM16, cat# 3163003B) Fludigim,
CD28 (clone L293, cat# 340975) BD Bioscience,
CD45RO (clone UCHL1, cat# 3165011B) Fludigim,
CD34 (clone 581, cat# 3166012B) Fludigim,
CD27 (clone O323, cat# 3167002B) Fludigim,
CD24 (clone ML5, cat# 3169004B) Fludigim,
CD20 (clone 2H7, cat# 3171012B) Fludigim,
IgM (clone MHM-88, cat# 3172004B) Fludigim,
CXCR4 (clone 12G5, cat# 3173001B) Fluidigm,
PD1 (clone EH12.2H7, cat# 3174020B) Fludigim,
CD11b (clone ICRF44, cat# 3209003B) Fluidigm,
Ki67 (clone B56, cat# 3168007B) Fludigim,
Tbet (clone 4B10, cat# 644825) Biolegend,
TNF (clone MAb11, cat# 3175023B) DVS Sciences,
CHI3L (clone EPR19078-157, cat# ab255864) abcam,

Validation

All antibodies were validated for use in the human immune cells using flow cytometry and subsequently mass cytometry.
